# Surgical treatment of post-traumatic luxation of rib heads with spinal cord compression in a cat

**DOI:** 10.1186/s13028-021-00619-6

**Published:** 2021-12-20

**Authors:** Jacek Cezary Sterna, Laura Rogowska, Beata Degórska, Jacek Sobczyński, Monika Łumińska

**Affiliations:** 1 Department of Small Animal Diseases and Clinic, Faculty of Veterinary Medicine, Warsaw University of Life Sciences, Nowoursynowska 159C, 02-776 Warsaw, Poland; 2 Klinika Weterynaryjna Bemowo, Powstańców Śląskich 101, 01-495 Warsaw, Poland

**Keywords:** Decompression, Feline medicine, Rib head, Spinal cord, Traumatology

## Abstract

**Background:**

Luxation of the rib head with intrusion into the intervertebral foramen seems to be rare in cats. Only one report has been published describing a cat with non-ambulatory paraparesis, which was managed conservatively. Here we report a case of rib head luxation that was managed surgically.

**Case presentation:**

A 4-year-old, female domestic shorthair cat with a two-week history of non-ambulatory paraparesis was presented at our clinic. Based on history and neurological examination, a diagnosis of thoracolumbar spinal cord lesion of traumatic origin was made. Computed tomography scanning revealed mild scoliosis, a luxation of the 3rd and 4th right rib heads and penetration into the spinal canal through intervertbral foramina. Surgical management using right dorsal approach to the spine was performed. The spinal cord was decompressed by cutting and removing of proximal ribs’ fragments by rotation and pulling out from the intervertebral foramina. The cat was ambulatory and paraparetic four weeks after surgery. Two years after surgery the cat regained functional gait, but ataxia remained.

**Conclusions:**

We report the first case of a surgical treatment of rib head luxation and intrusion into the spinal canal in a cat. The applied procedure resulted in an improvement of neurological signs.

## Background

In domestic animals**,** most ribs create two separate articulations with the vertebral column. The head of the rib is a part of a ball-and-socket *costovertebral* joint, which is characterized by unusually restricted mobility. The joint cavity is divided into two compartments by the intercapital ligament, which arises from the interarticular crest. Additional short, tight ligaments support the joint dorsally and ventrally. The *costotransverse* joint, in which the tubercle participates, is supported by a ligament that passes between the neck of the rib and the transverse process of the vertebra [1].

There has only been one report of luxation of rib head and dislocation into the intervertebral foramen in a cat [2]. In this case, the head of the right 9th rib was dislocated into the intervertebral foramen causing contusive injury of the spinal cord, without obvious compression. The lesion was associated with non-ambulatory paraparesis. The neurological status of the cat improved during hospitalization. Surgical treatment was considered, but it was not performed. After 6 weeks the cat was ambulatory and paraparetic. Six months after the treatment the owner did not notice any deviation from the normal movement of the animal [2].

In human medicine, luxation of a rib head into the spinal canal has been described mostly in cases of spinal deformities [3, 4]. There have been, however, reports of some cases of traumatic origin [3, 5].

## Case presentation

A 4-year-old female domestic shorthair cat weighing 3.5 kg was presented at Klinika Weterynaryjna Bemowo, Warsaw, Poland. According to the history, the cat fled from the owners’ house and was found, non-ambulatory and paraparetic, under the hood of a car 2 days later. The cat had been treated conservatively (with glucocorticoids, galantamine and vitamin B supplementation) by a referring veterinarian for two weeks, but its neurological condition did not improve. The cat showed a normal appetite and was consciously urinating and defecating.

During the initial visit to our clinic, approximately 2 weeks after the suspected injury, the cat was found to be non-ambulatory and had superficial wounds on the cheek and the chest wall, with the rest of the clinical examination findings being unremarkable. Further neurologic examination confirmed the paresis of the pelvic limbs and spinal pain in the thoracic region. There were increased patellar and tibialis cranialis reflexes together with a normal flexor reflex with the presence of a cross extensor reflex in both pelvic limbs. Propioceptive placing was present, but the postural reactions during hopping and wheelbarrowing were delayed. The cutaneous trunci reflex was present on the left side along the entire length and on the right side only caudally from L3. Nociception was present in both pelvic limbs. Serum biochemistry revealed increased levels of alanine transaminase and decreased levels of creatinine and blood urea nitrogen. There were no significant changes in the complete blood count. The initial diagnosis of a spinal cord lesion in the T3-L3 spinal column area of traumatic origin was made. Movement restriction was recommended together with the following medication: gabapentin (10 mg/kg PO) two times daily (Gabapentin—Teva, Teva) and meloxicam (0.1 mg/kg SC) once a day (Metacam, Boehringer Ingelheim), until imaging diagnostics could be performed.

**Fig. 1:**
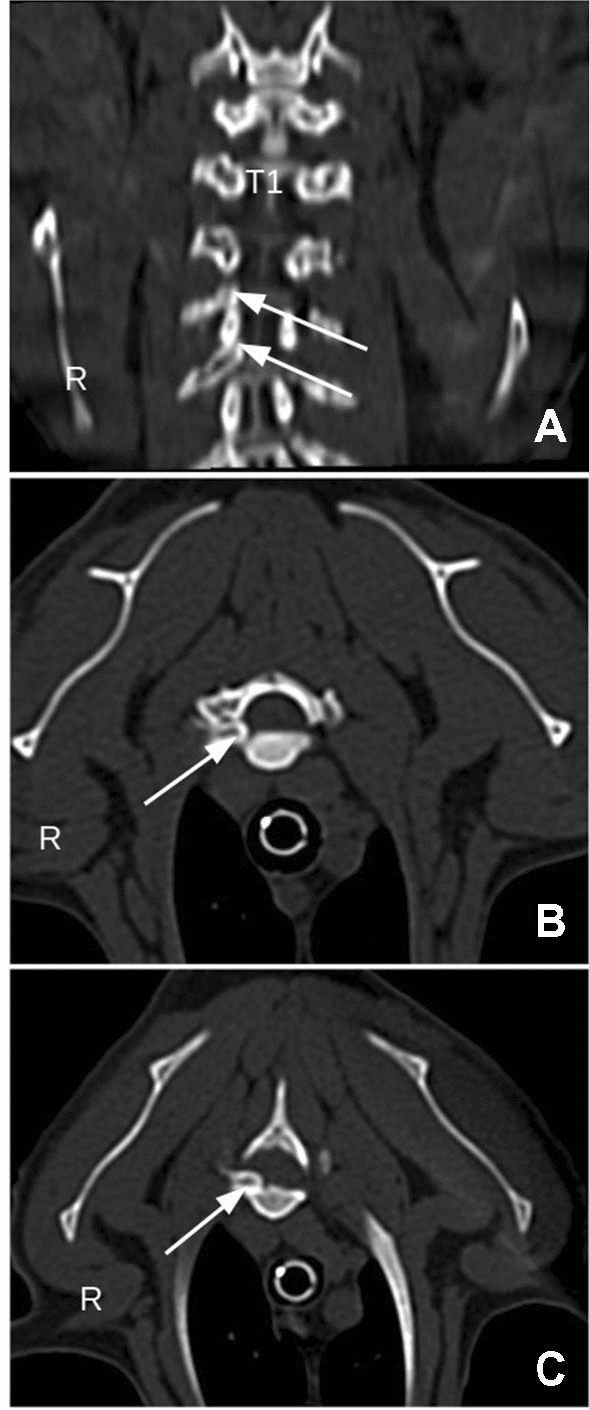
Computed Tomography findings. Coronal reconstruction of the thoracic region (**A**), Transversal plane at the level of the T2-T3 intervertebral space (**B**). Transversal plane at the level of the T3-T4 intervertebral space (**C**). Arrows point to the dislocated rib heads

Computed tomography (CT) scaning (16-slice multidetector, Siemens SOMATOM) of C1-S3 segment was performed three days later after premedication with dexmedetomidine (10 μg/kg IM) (Dexdomitor 0.5 mg/mL, Orion Pharma) and butorphanol (0.1 mg/kg IM) (Torbugesic, Zoetis) followed by anesthesia with propofol (1 mg/kg IV) (Propofol-Lipuro, B. Braun Melsungen). The CT examination revealed a luxation of the heads of the third and fourth ribs on the right side and dislocation into the vertebral foramina between T2 and T3 and, more severely, between T3 and T4. This dislocation caused spinal cord compression and potential compression of nerve roots. Additionally, mild thoracic scoliosis of T3-T6 with a Cobb angle of 20° was observed (Fig. 1). Taking into account the cat’s clinical status and the CT findings, a surgical intervention to decompress the spinal cord was decided.

The cat’s physical condition was classified as II out of IV according to the American Society of Anesthesiologists (ASA) scale. The cat was premedicated with dexmedetomidine (10 μg/kg IM) (Dexdomitor 0.5 mg/mL, Orion Pharma), midazolam (0.15 mg/kg IM) (Midanium, Polfa Warszawa), ketamine (4 mg/kg IM) (Bioketan, Vetoquinol) and methadone (0.3 mg/kg IM) (Comfortan, Dechra). Anesthesia was induced with propofol (1 mg/kg IV) (Propofol-Lipuro, B. Braun Melsungen) and maintained with isoflurane (Iso-Vet, Piramal) and with a constant-rate infusion of fentanyl (0.2 μg/kg/min) (Fentanyl WZF, Polfa Warszawa), lignocaine (10 μg/kg/min) (Lignocainum hydrochloricum WZF 2%, Polfa Warszawa) and ketamine (10 μg/kg/min) (Bioketan, Vetoquinol).

**Fig. 2:**
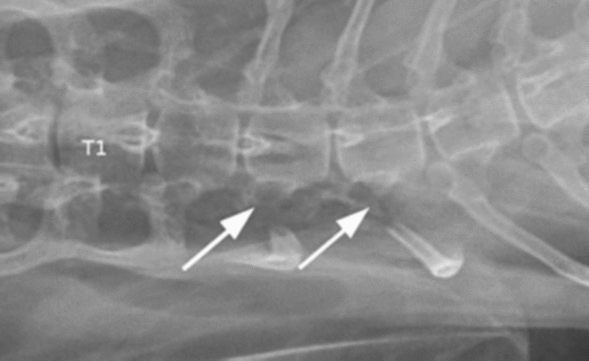
Postoperative ventrodorsal radiograph. Arrows point to the absence of the third and fourth rib heads on the right side

The cat was prepared for surgery *lege artis* and placed in sternal recumbency. The right dorsal approach was performed to the thoracic region to visualize the vertebral-costal articulation for the 3rd and 4th right ribs. An oxidized cellulose preparation (Pahacel, Altayar Medikal) or bipolar cautery was employed wherever hemorrhage was encountered. Access to the ribs was obtained starting from the 4th rib. Gentle traction appeared to be insufficient to reduce the luxation, and the rib could not be extracted from the intervertebral foramen. Therefore, an osteotomy was performed 1 cm distal to the rib head with a small Liston bone cutter. After the osteotomy, gentle traction with rotation allowed the reduction of the luxation, and this part of the 4th rib was removed. At this point, access to the 3rd rib was obtained. The proximal part of the rib was reached, cut, and luxation was reduced. It was removed in the same way for the 4th rib. The wound was closed by simple apposition of the paraspinal muscles and routine closure of subcutaneous tissue and skin. A ventrodorsal control radiograph was made immediately after the surgery and showed the excision of the correct rib heads (Fig. 2).

Postoperative analgesia consisted of placing a fentanyl patch (25 µg/h, for 72 h) (Durogesic, Janssen) and methadone (0.3 mg/kg IM) (Comfortan, Dechra) every four hours for the first 12 h after the procedure until the patch worked.

The cat was hospitalized for 24 h after surgery. During the hospitalization, neurological evaluation was performed in the morning and showed the deterioration of neurological signs. The cat was paraplegic and there was no nociception. At discharge in the evening, the cat was still paraplegic, but nociception recovered, as well as conscious urination. Continued treatment with gabapentin (10 mg/kg PO) twice a day (Gabapentin Teva, Teva) for 3–4 weeks, meloxicam (0.1 mg/kg PO) once a day (Metacam, Boehringer Ingelheim) for five days, and amoxicillin with clavulanic acid (14 mg/kg SC) once a day (Synulox, Haupt Pharma Latina) for seven days was prescribed.

The cat was reexamined four weeks later (about 6 weeks after suspected injury). Its condition had improved as it was now ambulatory although still paraparetic. It attempted to assume a standing position, and was able to take a few steps, but was still severely ataxic. Proprioceptive placing was delayed but present, as well as nociception. Physiotherapy was recommended; however, the owners did not decide to rehabilitate the cat professionally at a physiotherapy clinic. Instead, they chose to do it by themselves following a veterinarian’s recommendations, which involved passive and active exercises of the limbs, as well as support in standing up and walking attempts.

The long-term follow-up at the 4th and 17th month after surgery was conducted by a telephone conversation with the owners, as they lived far from the clinic. The owners were satisfied with the outcome, and the cat regained functional gait. Two years after the surgery, during a follow-up neurological examination, the cat showed functional gait, but ataxia remained. Proprioceptive positioning was present. Posture reactions during wheelbarrowing were normal. Spinal reflexes in pelvic limbs had slightly increased. There were no signs of spinal pain.

## Discussion and conclusions

At the time of the presentation, mild scoliosis was detected, which could have had an impact on the biomechanics of luxation and intrusion in the vertebral foramina of the 3rd and 4th right ribs, which were on the apex of the scoliotic curve. In human medicine, two mechanisms lead to the wedging of a rib head into the spinal canal: neurofibromatosis (NF-1) or trauma. The biomechanics behind this pathology are well described in kyphoscoliotic deformities associated with NF-1. In a posttraumatic case of scoliosis and rib head luxation into the spinal canal (in humans), scoliosis also has an acute angular curve, and the progression of this curve is a predisposing factor for rib head dislocation. The 2nd to 9th ribs articulate with two demifacets that are located adjacent and anterior to the neural foramen. Progression of a scoliotic curve increases the distance between the demifacets on the adjacent vertebrae in the convex apex of the curve and can potentially disrupt rib–vertebral articulation. Apical vertebral rotation may also play a role in disrupting rib–vertebral articulation [3]. As the scoliosis was of a higher degree in all the human cases [3, 4, 6] and since the biomechanics of the feline spine differ from those of the human spine, it is highly debatable whether this mechanism occurred in the reported case. Scoliosis could have occurred before the accident and could therefore be an incidental finding. This could possibly be a result of rib head luxation because *the costovertebral* joint, especially the rib head joint, plays an important role in the stability of the thoracic spine. This role of rib head joint has been investigated in a canine model [7].

Fractured rim of the intervertebral foramen, as an alternative phenomenon, may facilitate the rib head intrusion into the spinal canal, as previously reported in a cat [2].

Regarding resection of the dislocated rib head in humans during scoliosis corrective surgery, no clear consensus has been reached [8]. However, if there are symptoms of neurologic impairment, it is necessary to resect the compressing part [9]. In case of posttraumatic rib wedging in humans, however, neither the removal of rib heads nor the correction of scoliosis was performed due to the absence of neurological impairment and pain [3]. Similarly, for the cat with a spinal cord contusion, but without compression, it was not necessary to remove the dislocated rib head [2].

In the currently reported case, as the neurological signs were quite severe, surgical intervention was the method of choice. No correction of the scoliosis was performed, as it was of a low degree, and it was expected that it would subside after the removal of the rib heads. On the radiograph taken right after the surgery, scoliosis was still present. Unfortunately, there was no possibility for any long-term radiological follow-up.

The applied surgical treatment aimed at decompression of the spinal cord and improvement of neurological signs. These goals were eventually achieved. However, one might speculate whether performing hemilaminectomies, as is sometimes advised in humans, [9] or a gentler removal of the rib heads could lead to faster and/or more complete recovery. Gentler removal of the wedged rib heads would have been possible if a shorter time had elapsed from the suspected trauma to the surgery. However, over the two weeks, adhesions of the rib heads and the surrounding tissues developed. As a reason for the transient worsening of neurological status, i.e., paraplegia and the absence of nociception, apart from postsurgical edema and/or analgesic treatment, reperfusion injury should be taken into consideration. Several authors have suggested that decompression of chronic spinal cord lesions can lead to reperfusion injuries resulting in acute deterioration after surgery [10, 11]. Diagnostic of this phenomenon is based on magnetic resonance imaging (MRI) performed at the time of worsening—typical hyperintense signals on T2-weighted MRI in the area of the surgery. The usual treatment of reperfusion injuries in humans is administration of high doses of steroids and rehabilitation [12]. It is unclear why patients with osseous-associated cervical spondylomyelopathy and meningioma are more likely to experience early postoperative neurological deterioration than patients with other diagnoses. However, other disease processes, such as vertebral arch anomalies and spinal arachnoid diverticulum, which are characterized by chronic spinal cord compression, are not associated with an increased risk of early postoperative deterioration [13]. This may suggest that the chronicity of the disease process cannot entirely explain the occurrence of this complication. In conclusion, a possible explanation may be not only postsurgical edema, but also analgesic treatment taken in to consideration as a reason for problem with pain examination in cats [2], or a combination of these conditions. Due to the unavailability of MRI examination a more precise diagnosis cannot be obtained in the present case.

It is possible that a satisfactory neurological condition could have been achieved sooner if professional physiotherapy (such as neural stimulation, neuromuscular electrical stimulation, and hydrotherapy on a water treadmill, if the cat would tolerate it), had been started immediately after the surgery [14]. Unfortunately, a more direct comparison of the results of the surgical treatment reported here with the results of the conservative treatment reported in the literature could not be performed because of many differences (number of rib heads luxated, additional fractures, and various dates of follow-up examinations) between the two cases [2].

## Data Availability

The datasets used and/or analyzed during the current study are available from the corresponding author on reasonable request.

## Abbreviations

ASA: American Society of Anesthesiologists
CT: Computed tomography
L: Lumbar vertebra
MRI: Magnetic resonance imaging
NF-1: Neurofibromatosis type 1
T: Thoracic vertebra
